# Development and Evaluation of Photoprotective O/W Emulsions Containing Hydroalcoholic Extract of* Neoglaziovia variegata* (Bromeliaceae)

**DOI:** 10.1155/2017/5019458

**Published:** 2017-06-07

**Authors:** Raimundo Gonçalves de Oliveira-Júnior, Grasielly Rocha Souza, Christiane Adrielly Alves Ferraz, Ana Paula de Oliveira, Camila de Souza Araújo, Sarah Raquel Gomes de Lima-Saraiva, Silvio Alan Gonçalves Bomfim Reis, Talita Mota Gonçalves, Larissa Araújo Rolim, Pedro José Rolim-Neto, Francine Celise Siqueira César, Jackson Roberto Guedes da Silva Almeida

**Affiliations:** ^1^Universidade Federal do Vale do São Francisco, 56.304-205 Petrolina, PE, Brazil; ^2^Universidade Federal do Pernambuco, 50.670-901 Recife, PE, Brazil; ^3^Universidade de São Paulo, 14.040-900 Ribeirão Preto, SP, Brazil

## Abstract

*Neoglaziovia variegata *is a Bromeliaceae plant species widely found in Brazil with several pharmacological properties, including photoprotective activity. Although herbal-based active ingredients have been applied in cosmetic products, especially for skin treatment, its application in sunscreen formulations remains unexplored. The aim of this work is to evaluate the photoprotective effect of cosmetic formulations containing hydroalcoholic extract of* N. variegata* (Nv-HA). Initially, the phenolic and flavonoid total content of Nv-HA were determined. The photoprotective activity of Nv-HA was subsequently assessed using a spectrophotometric method. Nv-HA was incorporated in O/W emulsions in the presence or absence of synthetic filters and their photoprotective efficacy was evaluated by spectrophotometric SPF determination. Finally, the stability study of the formulations was performed through the freezing/defrosting method. Nv-HA showed significant phenolic and flavonoids content (61.66 ± 5.14 mg GAE/g and 90.27 ± 5.03 mg CE/g, resp.). Nv-HA showed SPF values of 5.43 ± 0.07 and 11.73 ± 0.04 for the concentrations of 0.5 and 1.0% (v/v), respectively. It was verified that Nv-HA potentiated the photoprotective effect of formulations containing only synthetic filters. Furthermore, the formulations have remained stable at the end of the preliminary stability study. According to the results, it was concluded that Nv-HA can be used as a chemical filter in cosmetic formulations.

## 1. Introduction

The incidence of skin cancer and photoaging induced by solar radiation grows significantly throughout the world. According to the World Health Organization (WHO), melanoma is the most common cancer and it has been considered a public health problem [1]. In tropical countries such as Brazil, the number of skin cancer cases has increased mainly due to its geographical location with more exposure to ultraviolet (UV) radiation [2].

The UV radiation (UVR) is divided into three ranges: UVA (320–400 nm), UVB (290–320 nm), and UVC (100–290 nm). Despite the fact that the atmospheric O_2_ and the ozone layer absorb UVC, UVR still can produce several deleterious effects in human skin including damage in DNA, RNA, and proteins. UVA and UVB may also induce the generation of Reactive Oxygen Species (ROS) that lead to the expression of cytokines, matrix metalloproteinases and mutations resulting in inflammation, signs of photoaging, and skin cancer [3–5].

The need to attenuate the negative effects of sunlight exposure, preventing skin damage and cancer, has triggered the development of cosmetic products containing chemicals that can absorb UV radiation (UV filters). To date, several topical sunscreen formulations have been commercialized and afford protection against both UVA and UVB radiation [6, 7].

Still, dermatological, toxicological, and environmental implications of currently available UV filters have opened up the door for new concepts in photoprotection with regard to the use of plant extracts [6–9].

Plant extracts have active ingredients able to scavenge free radicals, to protect the skin matrix over the inhibition of enzymatic degradation, or to promote collagen synthesis, affecting skin elasticity and hyperpigmentation [10]. Additionally, various phytochemicals possess aromatic structures, sometimes conjugated with carbon-carbon double bonds and/or carbonyl moieties, as phenolic compounds and flavonoids, which can effectively absorb photons and rapidly return to ground state, exactly as UV filters [6, 11].

The use of plant extracts in association with sunscreens formulations can fully protect the skin against UVR by protecting the skin matrix against oxidative stress and synergistically increase the Sun Protection Factor (SPF) as additional UV filters [10–12].

In this context, the Bromeliaceae family stands out for presenting species with an inexhaustible chemical and pharmacological potential. The phytochemistry of this family is characterized by the presence of steroids, terpenoids, and phenolic compounds, especially flavonoids [13]. In addition, some Bromeliaceae species have antioxidant and antibacterial [14] activities reported.

*N. variegata* is an endemic Bromeliaceae, popularly known as “caroá,” widely distributed in the northeast region of Brazil. Previous studies have shown that this plant has antimicrobial [14], antinociceptive [15], gastroprotective [16], and antioxidant and photoprotective activities [17, 18], probably related to the presence of phenolic and flavonoids. Despite its ethnopharmacological potential, the application of* N. variegata* extracts in sunscreens formulations remains unexplored, with no scientific reports or patent deposits (using Derwent World Patents Index database).

Although the photoprotective effect of extracts obtained from* N. variegata* has been investigated, there are no reports of the therapeutic properties of formulations containing active ingredients from this species. In this context, this is the first report on the development of pharmaceutical preparations containing* N. variegata* extract, as well as the evaluation of its stability through the measurement of quality parameters widely used in the cosmetics industry.

This study outlines the development of O/W emulsions containing hydroalcoholic extract of leaves from* N. variegata* as a synergistic active ingredient to increase the sunlight protection factor of commercially available synthetic UV filters.

## 2. Materials and Methods

### 2.1. Materials

The solvents were purchased from Synth® (Brazil), while the Folin-Ciocalteu reagent, DPPH (2,2-diphenyl-1-picrylhydrazyl), *β*-carotene, and linoleic acid were obtained from Sigma-Aldrich® (Brazil). The raw materials used to prepare the formulations were purchased from Mapric® (Brazil).

### 2.2. Plant Material

The leaves of* N. variegata* were collected in Petrolina, State of Pernambuco, Brazil, in January 2013 (coordinates S 08°59′16′′ and W 40°35′20′′). The samples were identified by a botanist from Centro de Recuperação de Áreas Degradadas da Caatinga (CRAD). A voucher specimen (6441) was deposited at the Herbarium Vale do São Francisco (HVASF) of the Universidade Federal do Vale do São Francisco (UNIVASF).

### 2.3. Preparation of Hydroalcoholic Extract (Nv-HA)

The extract of Nv-HA was performed according to the Brazilian Pharmacopoeia [19]. 200 g of dried and pulverized plant material was submitted to maceration with water-ethanol solution 56% (1000 mL). After 72 hours, 170 mL was collected and stored in amber container under refrigeration (4°C), while the remainder of the material was successively extracted with the same solvent solution. The extraction solution was concentrated on a rotary evaporator, at a maximum temperature of 60°C, until the volume of 30 mL, which was added to the previously 170 mL-extract solution, producing a total of 200 mL of hydroalcoholic fluid extract of the plant (Nv-HA). The final extract was considered 1 : 1 (weight/volume).

### 2.4. Total Phenolic Content of Nv-HA

Total phenolic content was determined using the Folin-Ciocalteu reagent, based on previously reported method [20]. An aliquot (40 *μ*L) of diluted Nv-HA (1 mg/mL) was added to 3.16 mL of distilled water and 200 *μ*L of the Folin-Ciocalteu reagent and mixed well. The mixture was shaken and allowed to stand for 6 min, before adding 600 *μ*L of sodium carbonate solution, as well as shaking to mix. The solutions were left at 20°C for 2 hours and the absorbance of each solution was determined using a spectrophotometer (Quimis, Brazil) at 765 nm against the blank and plot absorbance versus concentration. Total phenolic contents of the extracts (three replicates per treatment) were expressed as mg gallic acid equivalents per gram of sample (mg GAE/g) through the calibration curve with gallic acid. The calibration curve range was 50–1000 mg/L (*R*^2^ = 0.9928). All samples were performed in triplicate.

### 2.5. Total Flavonoid Content of Nv-HA

Total flavonoid content was determined using a previously described colorimetric method [21]. Briefly, 0.30 mL of the Nv-HA, or (+)-catechin standard solution, was mixed with 1.50 mL of distilled water in a test tube followed by addition of 90 *μ*L of 5% NaNO_2_ solution. After 6 min, 180 *μ*L of 10% AlCl_3_·6H_2_O solution was added and allowed to stand for another 5 min before 0.6 mL of 1 M NaOH was added. The mixture was brought to 330 *μ*L with distilled water and mixed well. The absorbance was measured immediately against the blank at 510 nm using a spectrophotometer (Quimis, Brazil) in comparison with the standards prepared similarly with known (+)-catechin concentrations. The results were expressed as mg of catechin equivalents per gram of extracts (mg CE/g) through the calibration curve with catechin (*R*^2^ = 0.9982). The calibration curve range was 50–1000 mg/L. All samples were analyzed in triplicate.

### 2.6. Photoprotective Activity In Vitro of Nv-HA: Determination of the Maximum Absorption Wavelength and Sun Protection Factor Spectrophotometric (SPF_spectrophotometric_)

The photoprotective efficacy of the extract was evaluated according to Violante and coworkers [22]. Aliquots of Nv-HA were suspended in distilled water at concentration of 0.5 to 1.0% (v/v). For maximum absorption wavelength (*λ*_max_) determination, spectrophotometric scanning of Nv-HA was performed at wavelengths between 260 and 400 nm, with intervals of 5 nm, using 1 cm quartz cell and ethanol as blank. Calculation of SPF was obtained according to [23]

(1) $$\begin{array}{c} {SPF}_{spectrophotometric}=CF\times \overset{320}{\underset{290}{\sum }}EE\left(\;\lambda \;\right)\times I\left(\;\lambda \;\right)\times Abs\left(\;\lambda \;\right), \end{array}$$

where EE(*λ*) is erythemal effect spectrum; *I*(*λ*) is solar intensity spectrum; Abs (*λ*) is absorbance of sunscreen product; CF is correction factor (=10). The values of EE × *I* are constant and previously determined [24]. Benzophenone-3 and quercetin (10 mg/L) were used as positive control.

### 2.7. Preparation of Emulsions

Anionic emulsion was selected for the development of photoprotective formulations, as shown in Table 1. The emulsion was prepared by the emulsification process [25], heating phases 1 and 2 separately to 75  ±  1°C. Then, phase 1 was mixed with phase 2, slowly and under constant agitation (1000 rpm). After formation of the emulsion, phases 3 and 4 were added at 40°C, maintaining the system under agitation until complete homogenization (500 rpm). The resulting emulsion was immediately subjected to quality control tests (resistance to centrifugation, pH, and relative viscosity) prior to the next stages of the study.

### 2.8. Preparation of Photoprotective Formulations

To prepare the photoprotective formulations, physical (zinc oxide and titanium dioxide) and chemical (octyl methoxycinnamate and benzophenone-3) synthetic filters were suspended in propylene glycol and later added to the emulsion previously obtained, as shown in Table 2. Similarly, Nv-HA was added to the emulsion at different concentrations, combined (F5 and F6) or not (F2 and F3) with the synthetic filters. The emulsion base was used as negative control (F1), whereas the formulation containing only synthetic filters (F4) was used as a positive control.

### 2.9. In Vitro Photoprotective Activity of Formulations: Determination of SPF_spectrophotometric_

The SPF of the prepared formulations was determined using an adapted version of previously described methodology [26]. Initially, 250 mg of each formulation was diluted in absolute ethanol. The solution was transferred to a volumetric flask of 25 mL, completing it until the final volume. The solution was placed in an ultrasonic apparatus for 5 minutes and filtered, discarding the first 5 mL. A 2.5 mL aliquot of the filtrate was transferred to a 25 mL flask and absolute ethanol was added. From the resulting solution, 5 mL was transferred to another 25 mL flask and completed with absolute ethanol to a final solution of 0.2 mg/L. To calculate the SPF_spectrophotometric_, the absorbance values of the final solution were measured in spectrophotometer (Quimis, Brazil) using *λ* 290–320 nm, 5 nm interval. The SPF_spectrophotometric_ was calculated based on three independent samples with the same composition (*n* = 3), using (1) [23].

### 2.10. Physicochemical Stability Tests

For the stability study, the freezing/defrosting method was used [25]. The formulations were subjected to organoleptic characteristics (sensorial analysis), pH value determination, viscosity, and SPF_spectrophotometric_ measures before (*T*0) and after six freezing/defrosting cycles (12th day, *T*12). Each cycle corresponds to 48-hour formulation storage using 4 ± 2°C/24 hours followed by 40 ± 2°C/24 hours. Centrifugation test was performed on 24 h after preparation of the formulations at 3000 rpm (Fanen, model 206 BL, Brazil) for 30 min at room temperature. The appearance, homogeneity, and organoleptic characteristics were evaluated by macroscopic analyses. The pH value (MS Tecnopon, model mPA-210, Brazil) was determined by inserting the electrode directly into the aqueous dilution 1 : 10 (w/v) of the sample. Viscosity determinations were obtained using a Brookfield viscometer (Quimis, model Q860 M21, Brazil) at 25 ± 2°C while SPF_spectrophotometric_ were measured as described before.

### 2.11. Statistical Analysis

The data were analyzed using the GraphPad Prism® version 5.0 and expressed as mean ± SD. Statistically significant differences were calculated by the application of Student's *t*-test or one-way analysis of variance (ANOVA) followed by Tukey's test, according to the case. Values were considered significantly different when *p* < 0.05.

## 3. Results

The total phenolic content by Folin-Ciocalteu reagent method resulted in Nv-HA = 61.66 ± 5.14 mg GAE/g whereas the total flavonoid content based on aluminum chloride complexation in colorimetric analysis produced Nv-HA = 90.27 ± 5.03 mg CE/g.

**Figure 1:**
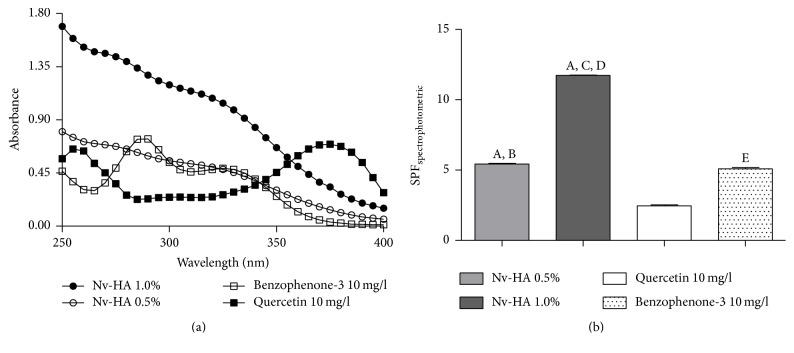
Spectrophotometric absorption spectra (a) and determination of the Sun Protection Factor (SPF) spectrophotometric (b) of NV-HA and standards (quercetin and benzophenone-3). Results are expressed as mean ± SD (*n* = 3), where ^A^(*p* < 0.05, Nv-HA 1.0% versus quercetin 10 mg/L), ^B^(*p* < 0.05, Nv-HA 0.5% versus quercetin 10 mg/L), ^C^(*p* < 0.05, Nv-HA 1.0% versus benzophenone-3 10 mg/L), ^D^(*p* < 0.05, Nv-HA 1.0% versus Nv-HA 0.5%), and ^E^(*p* < 0.001, benzophenone-3 10 mg/L versus quercetin 10 mg/L), according to one-way ANOVA followed by Tukey's test.

The in vitro photoprotective effect of Nv-HA determined by Mansur et al. (1986) evidenced the absorption in the UVB/UVA regions, as observed for the positive controls quercetin and benzophenone-3, suggesting a possible photoprotective activity (Figure 1).

In relation to SPF_spectrophotometric_, Nv-HA showed values of 5.43 ± 0.07 and 11.73 ± 0.04 for the concentrations of 0.5 and 1.0% (v/v), respectively (Figure 1), in a dose-response behavior. When compared to quercetin (SPF = 2.45 ± 0.13) and benzophenone-3 (SPF = 5.10 ± 0.15), Nv-HA 1.0% had the highest photoprotective effect.

**Figure 2:**
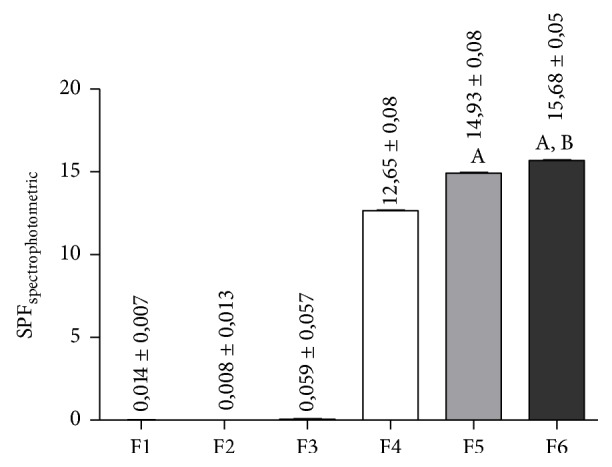
Determination of Sun Protection Factor (SPF) spectrophotometric of cosmetic formulations (F1–F6). Results are expressed as mean ± SD (*n* = 3), where ^A^(*p* < 0.05, F5 or F6 versus F4) and ^B^(*p* < 0.05, F5 versus F6), according to one-way ANOVA followed by Tukey's test.

After evaluation of the photoprotective activity of Nv-HA, the extract was incorporated in cosmetic formulations and its photoprotective efficacy was also investigated.  Figure 2 shows SPF values of all the formulations used in this study. It was revealed that only Nv-HA (F2 and F3) did not cause significant change in SPF values when compared to F1 (negative control). However, when associated with chemical and physical filters (F5 and F6), the extract was able to potentiate the photoprotective effect of the formulation containing only synthetic filters (F4), and this effect was more prominent in F6, which had higher percentage of Nv-HA.

The formulations presented visual appearance acceptable for cosmetic products throughout the stability study with no observed changes in color, odor, and appearance. Additionally, formulations showed no phase separation during the centrifugation test at any time of the study (*T*0 and *T*12), suggesting the emulsions was stable even when subjected to thermal stress in freezing/defrosting cycles.

In general, formulations had pH values between 6.0 and 8.0 immediately after sample manipulation. However the results also indicated the addition of synthetic filters has increased pH (pH F4, F5, and F6 > F1, F2, and F3). In case of the stability study, an increase of pH for F2 and F3 at *T*12 was verified.

**Figure 3:**
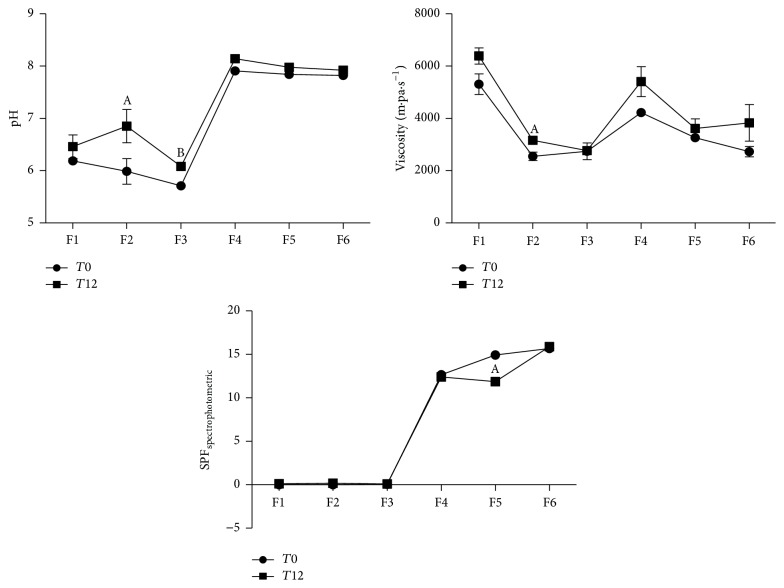
Evaluation of pH, viscosity (m·Pa·s^−1^), and Sun Protection Factor (SPF_spectrophotometric_) of photoprotective formulations (F1–F6) before (*T*0) and after (*T*12) the preliminary stability study. Results are expressed as mean ± SD, where ^A^(*p* < 0.05  *T*0 versus  *T*12) and ^B^(*p* < 0.05  *T*0 versus  *T*12), according to Student's *t*-test.

Formulations F2 and F3 showed lower viscosity when compared to F1. The same was observed for F5 and F6 in comparison with F4. Since Nv-HA has a high water content, its addition to the formulations may have contributed to the decrease of preparations viscosity. In contrast, F2 showed a significant increase in viscosity after the stability test, which can be explained by the loss of water due to the thermal stress. Despite the variation in viscosity of F2 during the thermal stress, the other formulations have no changes in viscosity during the stability study (Figure 3).

Another important step was to evaluate the SPF values of the samples before and after the preliminary stability study. The analysis of SPF before and after the preliminary stability study evidenced significant reduction in SPF values at *T*12 when compared to *T*0 to formulation F5 while F6 preserved it. The other formulations also showed no change in SPF_spectrophotometric_ values. Overall, these results confirm that formulations containing Nv-HA and synthetic filters had a satisfactory stability profile.

## 4. Discussion

Recent studies have evaluated the protective effect of natural products against damage caused by UVR. Plant extracts can protect the skin from UVA and UVB radiation in different ways. In most cases, these extracts have antioxidant, anti-inflammatory, immunomodulatory, antimutagenic, and photoprotective activities that can be justified by the presence of phenolic compounds and flavonoids [27].

The flavonoids are secondary metabolites with significant antioxidant and photoprotective potential. The protective effects of flavonoids are ascribed to their capacity to chelate metal [28], activate antioxidant enzymes [29], and stabilize free radicals and inhibit oxidases [30]. Furthermore, flavonoids can protect plants from solar UV radiation through distinct mechanisms of photoprotection, including UV absorption, direct and indirect antioxidant properties, and modulation of several signaling pathways [31].

In this sense, the Bromeliaceae family stands out for presenting species with significant content of phenolic compounds, especially flavonoids. Previous studies have shown that extracts and fractions from* N. Variegata* have high flavonoid content [14, 15]. Recently, the isolation of the first chemical constituent of* B. laciniosa*, 5,7-dihydroxy-3,3′,4′-trimethoxyflavone, an unprecedented flavonoid in the Bromeliaceae family, has been reported [32]. Furthermore, two flavonoids (isoquercetin and kaempferol-3-*O*-rhamnoside) and four phenolic acids (caffeic, protocatechuic,* p*-coumaric, and vanillic acids) were identified in different extracts of leaves and flowers from* N. variegata* by HPLC-DAD analysis [33]. In these reports, extracts and fractions were also evaluated in in vitro assays, showing good correlation between the flavonoid content and photoprotective and antioxidant activities [32, 33].

Here we investigated the photoprotective effect of a hydroalcoholic extract of* N. variegata*. After incorporation in formulations, Nv-HA did not present photoprotective activity. However, Nv-HA was able to potentiate the photoprotective effect of the formulations containing synthetic filters, promoting an increase in SPF_spectrophotometric_ values of samples (Figure 2). Although the test has been performed in vitro, it was demonstrated that this method correlates well with in vivo tests, because it relates the absorbance of the sample with the erythematogenic effect of radiation and intensity of light at specific wavelengths between 290 and 320 nm (UVB region) [22]. For this reason, these results suggest that Nv-HA can be used as an adjunct chemical filter in cosmetic sunscreen preparations reducing the concentration of synthetic filters and the risk of allergic reactions usually caused by excessive chemical filters, without compromising the photoprotective effect of the preparation.

In addition, the stability profile of developed formulations was evaluated. Stability is a parameter frequently described in analytical methods validation norms, but it is necessary to ensure the quality of phytocosmetics, from manufacturing to the expiry date. Several factors can interfere in stability of the product, including the manufacturing process, environmental, transport, and storage conditions, and formulation characteristics. The components of the formulation, whether active or not, can interfere in stability of the product, impairing its safety and efficacy [34]. In this study, satisfactory stability profile for formulations containing Nv-HA in different proportions was observed (Figure 3). Therefore, the use of Nv-HA has shown potential as an active ingredient to the development of skin care products with photoprotective properties, to be exploited in cosmetology. For this, other studies of safety and stability should be carried out until the product can be marketed.

## 5. Conclusions

The use of natural components in pharmaceutical preparations has increasingly been discussed in the research of new drugs and cosmetics centers. In this sense, this paper reports the development of cosmetic formulations containing a hydroalcoholic extract of leaves from* Neoglaziovia variegata*. Overall, the results demonstrated that the addition of Nv-HA potentiated the photoprotective activity of synthetic filters used commercially. Furthermore, the formulations have remained stable at the end of the preliminary stability study, in particular the formulation F6, preserving their photoprotective effect. These results demonstrate that the extract can be used as a chemical filter in cosmetic formulations, increasing the SPF of them.

## Figures and Tables

**Table 1:** General composition of the emulsion (emulsion base) used in the development of photoprotective formulations.

Component	Concentration% (w/w)	Phase
Mix of cetearyl alcohol and cetearyl sulfate sodium	6.00	1
Propylparaben	0.05	1
Methylparaben	0.15	2
Glycerin bidistilled	5.00	2
Propylene glycol	3.00	2
Imidazolidinyl urea	0.10	4
Distilled water	2.00	4
Cyclomethicone and dimethicone crosspolymer	1.00	3
Phenyl trimethicone	1.00	3
BHT	0.05	1
EDTA	0.05	2
Myristate isopropyl	5.00	1
Distilled water	s.q. 2000 mL	2

s.q: sufficient quantity to.

**Table 2:** Photoprotective formulations containing synthetic filters and/or Nv-HA in different percentages.

Component (%)	Formulations					
	F1	F2	F3	F4	F5	F6
Benzophenone-3	—	—	—	7	7	7
Octyl methoxycinnamate	—	—	—	5	5	5
Zinc oxide	—	—	—	2	2	2
Titanium dioxide	—	—	—	3	3	3
Nv-HA	—	5	10	—	5	10
Emulsion base	100	95	90	83	78	73

The percentages are expressed in v/w for Nv-HA and w/w for the other components.
